# Creation of a reference dataset of neck sizes in children: standardizing a potential new tool for prediction of obesity-associated diseases?

**DOI:** 10.1186/1471-2431-14-159

**Published:** 2014-06-21

**Authors:** Sherri L Katz, Jean-Philippe Vaccani, Janine Clarke, Lynda Hoey, Rachel C Colley, Nicholas J Barrowman

**Affiliations:** 1 Children’s Hospital of Eastern Ontario, Department of Pediatrics, Division of Respirology, 401 Smyth Road, Room W1444, Ottawa, Ontario K1H 8 L1, Canada; 2 University of Ottawa, Faculty of Medicine, Ottawa, Canada; 3 Children’s Hospital of Eastern Ontario, Department of Surgery, Division of Otolaryngology, Ottawa, Canada; 4 Statistics Canada, Health Statistics Division, Ottawa, Canada; 5 Children’s Hospital of Eastern Ontario Research Institute, Clinical Research Unit Ottawa, Ottawa, Canada

**Keywords:** Epidemiology, Sleep medicine, Neck circumference, Anthropometric measures, Obesity

## Abstract

**Background:**

Neck circumference (NC), is an emerging marker of obesity and associated disease risk, but is challenging to use as a screening tool in children, as age and sex standardized cutoffs have not been determined. A population-based sample of NC in Canadian children was collected, and age- and sex-specific reference curves for NC were developed.

**Methods:**

NC, waist circumference (WC), weight and height were measured on participants aged 6–17 years in cycle 2 of the Canadian Health Measures Survey. Quantile regression of NC versus age in males and females was used to obtain NC percentiles. Linear regression was used to examine association between NC, body mass index (BMI) and WC. NC was compared in healthy weight (BMI < 85^th^ percentile) and overweight/obese (BMI > 85^th^ percentile) subjects.

**Results:**

The sample included 936 females and 977 males. For all age and sex groups, NC was larger in overweight/obese children (*p* < 0.0001). For each additional unit of BMI, average NC in males was 0.49 cm higher and in females, 0.43 cm higher. For each additional cm of WC, average NC in males was 0.18 cm higher and in females, 0.17 cm higher.

**Conclusion:**

This study presents the first reference data on Canadian children’s NC. The reference curves may have future clinical applicability in identifying children at risk of central obesity-associated conditions and thresholds associated with disease risk.

## Background

Neck Circumference (NC) is an emerging marker of pediatric obesity, a rising epidemic and a major public health issue, with prevalence in Canada of 10% [1-3]. There is also some evidence that larger neck size may predict obesity [4,5] and conditions in children associated with being overweight or obese, including metabolic [6] and cardiovascular disease [7-9], as well as obstructive sleep apnea [10-14]. While body mass index (BMI) has traditionally been used to categorize individuals as healthy weight, overweight, or obese, it is becoming clearer that risk of associated diseases is determined by overweight/obesity [15], as well as where body fat is distributed. A larger NC, indicative of central body fat distribution, has been shown to be associated with cardiovascular and metabolic disease risk, as well as obstructive sleep apnea, in children and youth [6,8,14].

It is difficult, however, to establish thresholds of NC associated with disease risk in children, as normal neck size changes with age, sex and development. Age and sex-standardized NC values for children are therefore needed to better assist translation of this measurement into clinical practice.

To our knowledge, there are no reference data on neck circumference measurements in a large population-based sample of children in Canada. Some reference data is available from Germany [16] and Turkey; [4] however, these data sets may not be relevant for today’s North American population. Recent population-based data for Han children are also available, but in a narrower age range and homogeneity of ethnicity may limit generalization of results [17].

The Canadian Health Measures Survey (CHMS) is a large, nationally-representative survey which collected direct measures of NC in Canadian children and youth. Use of a healthy-weight, nationally representative sample of children to develop pediatric reference curves for NC is a strategy recommended by the World Health Organization in the development of growth curves, where a population with ideal health circumstances should be selected as the reference population [18]. This approach differs from that used in recent studies of NC which included overweight and obese children and youth, who may not be an ideal reference population [4,17]. The purpose of this study was to examine the association between NC and markers of adiposity in children, and to develop reference data on NC for the Canadian pediatric population, based upon data collected through the CHMS.

## Methods

### Data source

Cycle 2 of the Canadian Health Measures Survey (CHMS) covers the Canadian population aged 3 to 79 living in private dwellings. Residents of Indian Reserves or Crown lands, institutions, certain remote regions, and full-time members of the Canadian Forces are excluded. Approximately 96% of the Canadian population is represented.

Ethics approval for the survey was obtained from Health Canada’s Research Ethics Board [19,20]. Informed written consent was obtained from all respondents 14 years of age and older. Parents or guardians provided consent for children aged 3 to 13 and informed assent was obtained from the child.

Data for Cycle 2 of the CHMS were collected from 18 sites across Canada from September 2009 through December 2011. The survey consisted of two parts: 1) an in-home interview that collected information on socio-demographic characteristics and health behaviours; and 2) a subsequent visit to a mobile examination centre for a series of direct physical measurements, including various anthropometric and fitness tests, in addition to the collection of blood and urine samples [20].

Of the households selected, 75.9% agreed to participate. Within each responding household, one or two members were then selected to participate. Of those, 90.5% completed the household questionnaire, and 81.7% attended the mobile examination centre. The final response rate, after adjusting for the sampling strategy, was 55.5% [20]. The sample for this article is based on 1913 respondents aged 6 to 17 that completed the visit to the mobile examination centre and had valid NC, waist circumference (WC), and BMI data.

### Measures

NC and other anthropometric measurements such as height, weight, and WC were taken during the mobile examination centre visit, according to a detailed data collection protocol (CHMS Data User Guide) [20]. NC was measured using the most prominent portion of the thyroid cartilage as a landmark; the measurement was taken to the nearest 0.1 centimetres (cm) using a Gulick measuring tape (Fitness Mart, Gay Mills, USA) [21]. Height (cm) was measured using a Proscale M150 digital stadiometer (Accurate Technology Inc., Fletcher, USA), and weight (kg) was taken with a Mettler Toledo VLC with Panther Plus Terminal Scale (Mettler Toledo, Canada, Mississauga, Canada). WC (cm) was measured following the National Institutes of Health protocol, using the top of the iliac crest as a landmark. Body mass index was calculated for every respondent by dividing weight (kg) by height squared (m^2^). Age- and sex-specific cut-points from the Centres for Disease Control (CDC) were used to classify children and youth into two groups based on BMI: healthy-weight (BMI ≤85^th^ percentile), and overweight/obese (overweight: 85 < BMI ≤ 95^th^ percentile; obese: BMI >95^th^ percentile) [22].

All anthropometric measurements were taken by trained CHMS staff with a degree in Kinesiology and certification as Certified Exercise Physiologists® (http://www.csep.ca) and followed validated and standardized measurement techniques [20]. Staff performance was observed regularly and evaluated through the use of replicate measurements of all anthropometric data. Additionally, edits were incorporated into the data capture application to flag abnormal data entries outside of physiologic ranges, for review. Data was also verified during the validation process where the results are compared to similar datasets (e.g. Cycle 1), and/or reviewed by external experts to identify and remove invalid data prior to the data release. Detailed quality assurance and quality control procedures for data collection and processing were followed [20].

### Statistical analysis

Descriptive statistics were produced by sex, age (6 – 10, 11 – 14, and 15 – 17 years) and BMI group for height, weight, WC, and NC. The distribution of continuous variables was examined using percentile plots. Mean NC by age, sex and BMI category were also calculated, along with 95% confidence intervals. T-tests by sex, age and BMI group were used to compare mean anthropometric values between healthy-weight and overweight/obese individuals.

To examine the association between NC and other markers of overweight/obesity, linear regression was used to model (a) NC versus BMI, adjusted for age and (b) NC versus WC, adjusted for age. This was done for males and females separately, and also using an interaction by sex. P-values and adjusted r-square statistics were used to determine the significance and explanatory power of the model. Two-sided significance was set at p < 0.05.

In order to create a reference dataset for NC, only the healthy-weight sample was considered. This classification of healthy weight or overweight/obese was chosen to ensure that the sample used to develop the reference growth curves represented an “ideal healthy population”, as recommended by the World Health Organization [18]. For males and females separately, quantile regression was used to model NC versus age. Quantile regression allows flexible modeling of the conditional distribution of the response variable. Since it does not make distributional assumptions about the response, inferences are quite robust to outliers in the response observations [23]. Furthermore, quantile regression has been found to yield similar estimates to the LMS method but quantile regression requires fewer distributional assumptions and is more flexible than LMS [24]. Polynomial fits using integer powers of age were used. The order of the polynomial was increased until none of the Wald tests [23] for individual quantiles were statistically significant. For both males and females, a quantile regression model using a 4^th^-order polynomial in age was ultimately fitted to NCs. Reference curves were constructed for the 95^th^, 90^th^, 75^th^, 50^th^, 25^th^ and 5^th^ percentage points, chosen as they correspond to percentage points on the CDC growth charts, [25]. We were unable to reliably estimate the extremes at the 97% and 3% points, given our sample size.

Finally, the sensitivity and specificity of various NC percentile cut-off values for predicting a BMI of overweight or obese (BMI >85^th^ percentile) were determined from the quantile regression model fit. A receiver operator characteristic (ROC) curve was plotted in order to determine the most appropriate cut-off point for NC in a clinical setting. Note that since NC percentiles were obtained from a sample with BMI < 85^th^ percentile, the specificity is almost the same as the NC threshold.

All analyses were conducted with SAS Version 9.2 and SUDAAN Version 10 and were based on weighted data using the CHMS sample weights. To account for the survey design of the CHMS, standard errors, coefficients of variation and 95% confidence intervals were estimated using the bootstrap technique and specifying 13 denominator degrees of freedom in the SUDAAN procedure statements [20].

## Results

The total sample size was 1913, consisting of 936 females and 977 males. Age and anthropometric characteristics of the sample are presented in Table 1 by age, sex and BMI group. For all age and sex groups, weight, WC, BMI and NC were significantly larger in overweight/obese individuals compared to individuals who were neither overweight nor obese (Table 1).

**Table 1:** Characteristics of the weighted analyzed sample (n = 1,913), mean (95% CI) by weight category, age group, and sex. (Source: 2009–2011 Canadian health measures survey)

	**6 to 10 years**		**11 to 14 years**		**15 to 17 years**		**All ages**	
	**Males**	**Females**	**Males**	**Females**	**Males**	**Females**	**Males**	**Females**
	**Healthy weight**							
**Sample size**	306	328	240	240	152	143	698	711
**Age (yr)**	7.9 (7.7 – 8.2)	8.1 (7.8 – 8.3)	12.6 (12.3 – 12.9)	12.4 (12.2 – 12.6)	15.8 (15.7 – 16)	16 (15.7 – 16.2)	11.9 (11.5 – 12.3)	11.7 (11.5 – 11.9)
**Height (cm)**	130.7 (128.6 – 132.8)	131 (129.2 – 132.8)	157.6 (153.3 – 161.9)	154.9 (153.2 – 156.5)	174.2 (171.7 – 176.7)	163.3 (161.7 – 164.9)	152.7 (149.4 – 156.1)	147.8 (146.3 – 149.4)
**Weight (kg)**	27.8 (26.5 – 29)	28 (26.7 – 29.3)	46.1 (42.6 – 49.5)	44.7 (43.3 – 46.1)	62.9 (60.7 – 65)	55.5 (53.6 – 57.4)	44.4 (42 – 46.8)	41.1 (39.8 – 42.5)
**Waist circumference (cm)**	56.6 (55.3 – 57.9)	56.4 (55.2 – 57.6)	66.2 (64.3 – 68.1)	65.6 (64.6 – 66.7)	72.7 (71.3 – 74.2)	71.7 (69.9 – 73.5)	64.7 (63.5 – 65.8)	63.7 (62.6 – 64.8)
**Body mass index (kg · m-2)**	16.1 (15.7 – 16.6)	16.1 (15.8 – 16.5)	18.2 (17.6 – 18.7)	18.5 (18.1 – 19)	20.6 (20.2 – 21.1)	20.8 (20.3 – 21.3)	18.2 (17.9 – 18.5)	18.2 (17.9 – 18.5)
**Neck circumference (cm)**	26.8 (26.4 – 27.2)	26 (25.8 – 26.2)	30.8 (30.1 – 31.4)	28.9 (28.6 – 29.1)	34.7 (34.3 – 35.1)	30.4 (30.1 – 30.6)	30.5 (30–31)	28.2 (28 – 28.4)
	**Overweight/obese**							
**Sample size**	123	100	90	81	66	44	279	225
**Age (yr)**	8.3 (7.7 – 8.8)	8.3 (8 – 8.5)	12.3 (12.1 – 12.6)	12.7 (12.4 – 13)	16.1 (15.7 – 16.5)	15.8 (15.5 – 16.1)	11.5 (10.6 – 12.3)	12.2 (11.5 – 12.9)
**Height (cm)**	135.5 (130.8 – 140.3)	136^†^ (134.6 – 137.3)	162 (159.3 – 164.7)	161^†^ (157.8 – 164.1)	175.2 (172.2 – 178.1)	163.4 (158.2 – 168.6)	153.7 (148.4 – 159)	153.8^†^ (150.5 – 157.2)
**Weight (kg)**	42.4^†^ (37.9 – 46.9)	40.2^†^ (38.5 – 41.8)	68.4^†^ (63 – 73.9)	65.6^†^ (63.7 – 67.6)	88.6^†^ (81.9 – 95.3)	80.4^†^ (71.9 – 88.9)	62.1^†^ (56.1 – 68.1)	61.7^†^ (57.7 – 65.8)
**Waist circumference (cm)**	73.2^†^ (68.6 – 77.9)	71.4^†^ (69 – 73.9)	86.1^†^ (82 – 90.3)	83^†^ (81 – 84.9)	94.7^†^ (91.2 – 98.2)	91.6^†^ (86.3 – 96.9)	82.6^†^ (79.4 – 85.8)	81.7^†^ (79.5 – 84)
**Body mass index (kg · m-2)**	22.7^†^ (21.5 – 23.9)	21.5^†^ (20.6 – 22.3)	25.8^†^ (24.4 – 27.1)	25.3^†^ (24.5 – 26)	28.9^†^ (27.2 – 30.5)	29.9^†^ (28.4 – 31.5)	25.2^†^ (24.2 – 26.2)	25.4^†^ (24.5 – 26.3)
**Neck circumference (cm)**	29.9^†^ (28.9 – 30.9)	28.4^†^ (28–28.8)	33.9^†^ (32.6 – 35.2)	32.5^†^ (32.1 – 33)	38.4^†^ (37.3 – 39.6)	33.8^†^ (32.7 – 34.9)	33.3^†^ (32.1 – 34.5)	31.6^†^ (30.9 – 32.3)

^†^Significantly different from estimate for the Healthy group for the same age group and sex (p < 0.0001).

Results of the age-adjusted linear regressions examining the relationship between NC and BMI, and between NC and WC are presented in Table 2, stratified by sex and by healthy weight, or overweight/obesity. In each case the relationship is statistically significant (p < 0.0001). The introduction of an interaction with sex revealed that increases in WC or BMI in males are associated with greater increases in NC than in females (p < 0.0001 in all cases).

**Table 2:** Regression coefficients for neck circumference versus body mass index and waist circumference, age adjusted, by sex and weight category

**Variable**	**Sex**	**Beta (95% CI)**	**R**^**2**^	**p-value (beta)**
**Males**				
**BMI (kg/m**^**2**^**)**	Healthy-weight	0.75 (0.62 – 0.88)	0.88	<0.0001
	Overweight/obese	0.46 (0.38 – 0.54)	0.88	<0.0001
**Waist circumference (cm)**	Healthy-weight	0.24 (0.18 – 0.3)	0.86	<0.0001
	Overweight/obese	0.16 (0.13 – 0.18)	0.87	<0.0001
**Females**				
**BMI (kg/m**^**2**^**)**	Healthy-weight	0.42 (0.37 – 0.47)	0.8	<0.0001
	Overweight/obese	0.37 (0.26 – 0.48)	0.72	<0.0001
**Waist circumference (cm)**	Healthy-weight	0.15 (0.12 – 0.17)	0.8	<0.0001
	Overweight/obese	0.15 (0.13 – 0.17)	0.75	<0.0001

**Figure 1:**
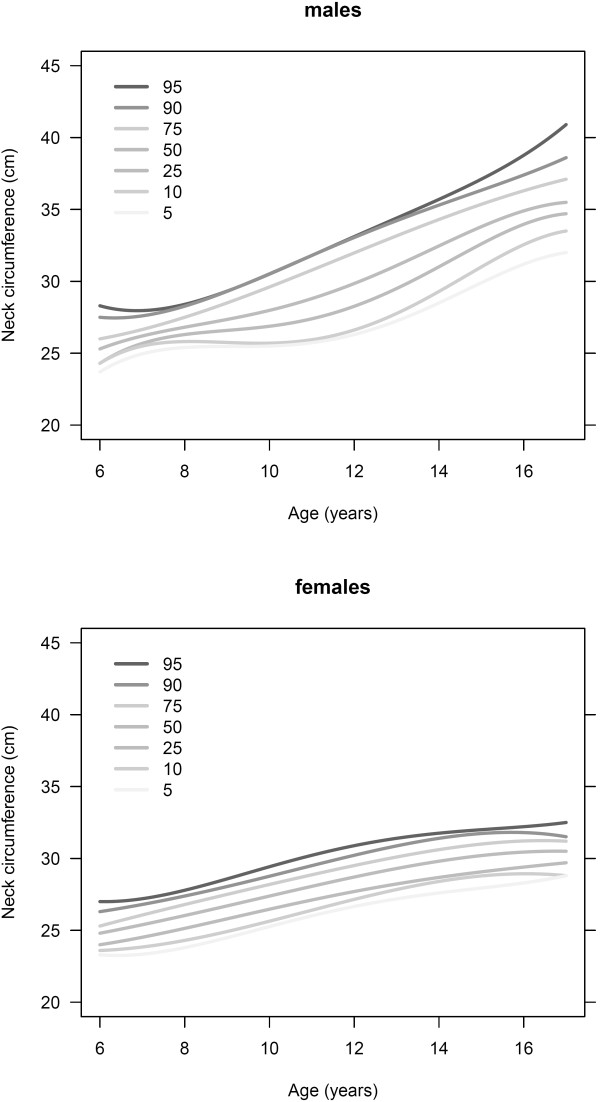
Selected quantile regression curves for males and females, household population aged 6 to 17 years, Canada, 2009 to 2011.

Table 3 shows the percentiles of NC estimated from the quantile regression model, by sex and age, along with 95% confidence intervals, for the reference, healthy-weight population. NC percentile estimates from the model tended to be larger with increasing age, and tended to be higher in males compared to females. The range of NC (5^th^ to 95^th^ estimates) in males was higher than in females, particularly for those approximately age 10 years and older. Curves of NC percentile estimates from the model by age and sex are displayed graphically in Figure 1.

**Table 3:** Quantile estimates for neck circumference (cm) by age (years)

**(a) Healthy-weight males**							
**Age**	**95th**	**90th**	**75th**	**50th**	**25th**	**10th**	**5th**
**6**	28.3 (27.3 – 29.3)	27.5 (26.2 – 28.8)	26 (24.7 – 27.3)	25.3 (24.7 – 25.9)	24.3 (23.6 – 25)	24.3 (23.4 – 25.2)	23.7 (22.5 – 24.9)
**7**	28 (27.4 – 28.6)	27.6 (27 – 28.2)	26.7 (25.8 – 27.5)	26.2 (25.6 – 26.8)	25.7 (25.3 – 26.1)	25.5 (25.1 – 25.9)	25 (24.4 – 25.5)
**8**	28.4 (28 – 28.8)	28.3 (27.8 – 28.7)	27.5 (26.7 – 28.3)	26.8 (26.3 – 27.4)	26.3 (25.8 – 26.8)	25.8 (25.4 – 26.2)	25.4 (24.9 – 25.9)
**9**	29.3 (28.9 – 29.7)	29.3 (28.7 – 29.9)	28.5 (27.7 – 29.3)	27.3 (26.9 – 27.8)	26.6 (25.9 – 27.2)	25.7 (25.2 – 26.3)	25.5 (24.9 – 26)
**10**	30.5 (30.1 – 30.9)	30.5 (29.9 – 31.1)	29.6 (28.9 – 30.3)	28 (27.6 – 28.4)	26.9 (26.1 – 27.7)	25.7 (24.8 – 26.6)	25.5 (24.8 – 26.2)
**11**	31.8 (31.4 – 32.2)	31.8 (31.3 – 32.3)	30.8 (30 – 31.5)	28.8 (28.4 – 29.2)	27.4 (26.6 – 28.2)	25.9 (24.9 – 27)	25.7 (24.8 – 26.7)
**12**	33.1 (32.7 – 33.5)	33 (32.5 – 33.6)	32 (31.2 – 32.8)	29.8 (29.3 – 30.4)	28.3 (27.5 – 29)	26.6 (25.5 – 27.8)	26.3 (25.3 – 27.3)
**13**	34.4 (33.8 – 35)	34.2 (33.6 – 34.8)	33.2 (32.3 – 34.1)	31.1 (30.5 – 31.7)	29.5 (28.9 – 30.1)	27.8 (26.5 – 29)	27.2 (26.2 – 28.3)
**14**	35.7 (34.9 – 36.5)	35.3 (34.6 – 36)	34.3 (33.3 – 35.3)	32.5 (31.8 – 33.2)	31 (30.5 – 31.5)	29.3 (27.9 – 30.7)	28.5 (27.3 – 29.7)
**15**	37.1 (36.3 – 37.9)	36.3 (35.6 – 37)	35.4 (34.3 – 36.4)	33.8 (33.1 – 34.5)	32.6 (32.1 – 33.1)	31 (29.5 – 32.5)	29.9 (28.7 – 31.1)
**16**	38.8 (37.7 – 39.8)	37.4 (36.1 – 38.6)	36.3 (35.5 – 37.1)	34.9 (34.4 – 35.4)	34 (33.5 – 34.5)	32.6 (31.3 – 33.8)	31.2 (30 – 32.4)
**17**	40.9 (38.1 – 43.7)	38.6 (35.7 – 41.5)	37.1 (35.5 – 38.7)	35.5 (34.3 – 36.6)	34.7 (34.2 – 35.2)	33.5 (31.9 – 35.1)	32 (29.5 – 34.5)
**(b) Healthy-weight females**							
**Age**	**95th**	**90th**	**75th**	**50th**	**25th**	**10th**	**5th**
**6**	27 (26.4 – 27.6)	26.3 (25.2 – 27.4)	25.3 (24.9 – 25.7)	24.8 (24.3 – 25.3)	24 (23.7 – 24.3)	23.6 (23.1 – 24.1)	23.3 (22.3 – 24.3)
**7**	27.2 (26.7 – 27.7)	26.8 (26.2 – 27.4)	26.1 (25.7 – 26.5)	25.4 (25 – 25.8)	24.5 (24.2 – 24.8)	23.8 (23.2 – 24.5)	23.3 (22.7 – 24)
**8**	27.8 (27.2 – 28.3)	27.4 (26.9 – 27.9)	26.8 (26.4 – 27.2)	26 (25.7 – 26.4)	25.1 (24.8 – 25.5)	24.3 (23.7 – 24.9)	23.8 (23.2 – 24.4)
**9**	28.6 (28.1 – 29.1)	28 (27.6 – 28.4)	27.5 (27.2 – 27.8)	26.7 (26.5 – 26.9)	25.8 (25.5 – 26.1)	24.9 (24.6 – 25.2)	24.5 (24.1 – 24.9)
**10**	29.4 (28.9 – 29.9)	28.8 (28.4 – 29.1)	28.2 (27.9 – 28.4)	27.4 (27.1 – 27.6)	26.5 (26.2 – 26.7)	25.6 (25.3 – 26)	25.3 (24.8 – 25.7)
**11**	30.2 (29.7 – 30.8)	29.5 (29.2 – 29.8)	28.9 (28.6 – 29.1)	28.1 (27.7 – 28.4)	27.1 (26.8 – 27.4)	26.4 (25.8 – 27)	26 (25.4 – 26.6)
**12**	30.9 (30.3 – 31.5)	30.2 (29.8 – 30.6)	29.5 (29.2 – 29.8)	28.7 (28.3 – 29.1)	27.7 (27.4 – 28)	27.2 (26.5 – 27.8)	26.7 (26 – 27.3)
**13**	31.4 (30.7 – 32.1)	30.9 (30.5 – 31.2)	30.1 (29.7 – 30.5)	29.3 (28.9 – 29.7)	28.2 (27.9 – 28.6)	27.8 (27.3 – 28.4)	27.2 (26.6 – 27.8)
**14**	31.8 (31.1 – 32.5)	31.4 (31.1 – 31.7)	30.6 (30.1 – 31.1)	29.8 (29.5 – 30.1)	28.7 (28.2 – 29.1)	28.4 (27.9 – 28.9)	27.6 (27.1 – 28.1)
**15**	32 (31.4 – 32.6)	31.7 (31.4 – 32.1)	31 (30.4 – 31.6)	30.2 (29.9 – 30.5)	29.1 (28.5 – 29.6)	28.8 (28.2 – 29.4)	27.9 (27.5 – 28.4)
**16**	32.2 (31.3 – 33.1)	31.8 (31.5 – 32.1)	31.2 (30.7 – 31.7)	30.4 (30.2 – 30.6)	29.4 (28.9 – 29.9)	28.9 (28.4 – 29.5)	28.3 (27.9 – 28.7)
**17**	32.5 (29.9 – 35.1)	31.5 (30.8 – 32.2)	31.2 (30.9 – 31.5)	30.5 (30.1 – 30.9)	29.7 (29 – 30.4)	28.8 (28.3 – 29.3)	28.8 (28.4 – 29.2)

**Figure 2:**
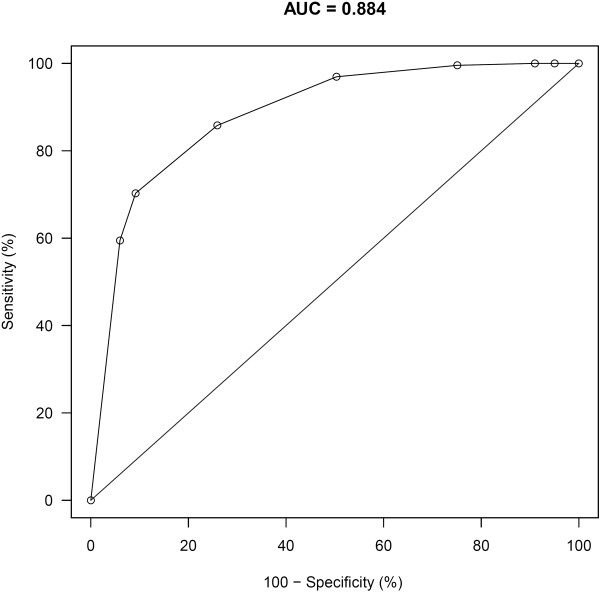
Receiver Operator Curve of Neck Circumference Percentile by BMI Percentile.

Results of the sensitivity and specificity analysis of NC percentile and BMI are presented as a receiver operator characteristic curve in Figure 2. The area under the ROC curve was 0.88 suggesting NC is useful in predicting overweight and obesity. For example, a NC value above the 50^th^ percentile for this sample yields a sensitivity of 97% and specificity of 50% for predicting BMI above the 85^th^ percentile.

## Discussion

The purpose of this study was to create a reference dataset of NC by age and sex using quantile regression analysis in a sub-sample of healthy-weight children. Using the reference dataset, we found that a NC above the 50^th^ percentile is a sensitive predictor of overweight/obesity (BMI > 85^th^ percentile).

The results of this study provide age and sex-standardized reference values of NC that can be used in future studies to examine the predictive ability of a NC threshold for overweight and obesity-associated co-morbidities. This may be of particular interest for prediction of obstructive sleep apnea in older children, since its etiology is specifically linked to fat distribution in the neck in adults and is likely similar in older youth [26,27]. Furthermore, measuring NC may have some advantages over measurements of generalized adiposity (BMI) and WC, which has been shown to be challenging to measure in children [28,29].

For both males and females, NC increases with age. In both sexes, variability in NC increases with increasing age and there is divergence of the quantile regression curves, as seen in Figure 1. This is particularly evident at age 11–14 years in females and 15–17 years in males. Furthermore, in females, fitted quantiles of NC exhibit the onset of a plateau between the ages of 13 and 16 years. The increased variability in NC may reflect variable onset of puberty, which is associated with significant somatic growth. In girls, the onset of puberty typically occurs at age 10–11, with the growth spurt between 11 and 12 years, whereas in boys, onset of puberty is slightly later and the growth spurt typically occurs at 13–14 years of age [30]. These age ranges for typical pubertal onset coincide with the increased variability in NC, supporting this hypothesis.

The NC values reported in this study differ from most of the existing literature, which relied on raw NC, which is not standardized for age and sex. This study also included only healthy weight individuals, an ideal reference population [18], unlike previous studies [5,29], which included overweight/obese children [4,29] or children in a narrower age range [6,8]. A Turkish population-based study derived similar NC percentiles for boys, but NC values for girls tended to be lower in our study, a finding which may be explained by the exclusion of overweight/obese children in our sample [4]. Nonetheless, correlation between NC, BMI and WC are similar to that previously reported in an elective surgical population [5].

The reference values determined in this study will enable clinicians to identify children with NCs that are different from healthy-weight Canadian children of the same age and sex. Further studies are needed to determine whether elevated NC is a predictor of other co-morbid health conditions. Thresholds of NC percentiles used to identify those at higher risk of other health conditions may vary, however, according to the setting in which they are used. NC percentile above the 50^th^ percentile provides high sensitivity for predicting those with BMI above the 85^th^ percentile (Figure 2) and may ultimately be demonstrated to be a good screening test, which would assist primary care providers in prioritizing referrals for diagnosis and treatment of obstructive sleep apnea or cardiovascular disease. When allocating resources for less widely available tests, such as polysomnography to evaluate obstructive sleep apnea, however, a threshold of NC above the 75^th^ percentile, which yields a sensitivity of 86% and specificity of 74%, may be more useful. Further research about how enlarged NC is related to co-morbidities of obesity, will allow refinement of this model.

Although we have a high degree of confidence in the data quality of this analysis, as the CHMS uses rigorous standards for measurement and analysis, this study does have some limitations. First, the overall response rate of the CHMS was 55.5%. Adjustments were made to the sampling weights to compensate for this. Despite the response rate, the sample size obtained was still large and representative enough for the creation of reference curves. Second, a well-known issue in growth curve modelling concerns “edge effects”: estimates are least precise at the oldest and youngest ages, and flexible curves may exhibit undesirable behaviour near these boundaries [31]. The confidence intervals in Table 3 show that at the lower and upper ages, the estimated quantiles are less precise. The estimated quantiles near these extremes should be treated with caution. Despite these limitations however, to the best of our knowledge, this is the first study to use a validated NC measurement in a population-based study of Canadian children and youth to construct reference curves.

## Conclusion

In conclusion, this study demonstrates that NC increases with age, BMI and WC in children and youth aged 6 to 17. Furthermore, reference values of NC for healthy-weight children and youth in a Canadian population have been determined. Elevated NC percentile may ultimately prove to be a useful adjunct to BMI or WC in identifying children and youth who are at risk for overweight and obesity-related conditions such as obstructive sleep apnea, although future work is needed to determine NC cut-offs or percentiles that correspond to increased health risk in children. The work presented here represents the first step towards achieving that goal.

## Abbreviations

BMI: Body mass index; CDC: Centres for Disease Control; CHMS: Canadian Health Measures Survey; CI: Confidence interval; NC: Neck circumference.

## Competing interests

The authors declare that they have no competing interests.

## Authors’ contributions

Drs. SLK, J-PV and RCC were responsible for the study conception and design, as well as the interpretation of the data and manuscript preparation. Dr. NJB and Ms. JC were responsible for the data analysis and assisted with both study design and data interpretation. Ms. LH was responsible for data collection and oversaw the training of data collectors, as well as contributing to the study design and manuscript preparation. All authors have had input into the manuscript and have approved the final version.

## Pre-publication history

The pre-publication history for this paper can be accessed here:

http://www.biomedcentral.com/1471-2431/14/159/prepub
